# A Retrospective Study: Do Hospital Menus Carry a Risk of Malnutrition?

**DOI:** 10.1002/fsn3.70669

**Published:** 2025-07-29

**Authors:** Beyza Mendes, Ayse Gunes Bayir, Ayse Semra Aksoy, Ozlem Toluk

**Affiliations:** ^1^ Department of Nutrition and Dietetics, Faculty of Health Sciences Bezmialem Vakif University Istanbul Türkiye; ^2^ Department of Food Hygiene and Technology, Faculty of Veterinary Medicine Kastamonu University Kastamonu Türkiye; ^3^ Department of Biostatistics and Medical Informatics, Faculty of Medicine Bezmialem Vakif University Istanbul Türkiye

**Keywords:** hospital menus, malnutrition, nutrient intake, nutrient profile, nutrients

## Abstract

Malnutrition is preventable, and increasing investment in food and nutrient care for patients can save lives. A retrospective study evaluated the adequacy levels of the menus served in a hospital by making meal‐based seasonal comparisons and examined the potential of these menus to pose a risk of malnutrition. In this context, the nutritional profile regarding energy, macro and micronutrients, and fiber was revealed. The results were compared with the Food and Drug Administration (FDA), the European Food Safety Authority (EFSA) and Türkiye Dietary Guideline‐2022 (TDG‐2022) recommendations. The nutrient profile of the menus was assessed by the Nutrient Rich Food NRF20.3 index (NRF20.3) and Limiting Nutrients 3 (LIM3) subscores. A one‐year dataset obtained from a hospital menu includes three meals (breakfast, lunch, and dinner) with various menus (general, diabetic, and gluten‐free) were examined, and the results of their nutrient profile were significantly different (*p* = 0.001). The NRF20.3 score for all menus and meals for a year was insignificant, while LIM3, the general menus for breakfast in spring, were higher than those of other menus (*p* = 0.001). The energy content of the general menus was between 1,621 and 1,663 kcal, which is high according to TDG‐2022, instead of EFSA and FDA. Daily potassium, calcium, magnesium, and fiber of the menus were low according to TDG‐2022 and EFSA, which is in accordance with including more meat and fewer vegetables, fruits, and dairy groups seasonally. Sodium and phosphorus in the menus were high. It was seen that the nutrient profile of the hospital menus examined in the study was not compatible with TDG‐2022. The menus' high energy, fat, saturated fat, sodium, and low calcium and fiber content lead to imbalances in nutrient intake. This situation can lead to nutrient deficiencies in hospitalized individuals. However, when the amount of protein in the menus is considered, it is seen that hospital menus pose a risk of malnutrition for men while being sufficient for women.

## Graphical abstract

This study aimed to assess the adequacy levels of menus served in a hospital by making meal‐based seasonal comparisons. In this context, the nutritional profile regarding energy, macro‐ and micronutrients, and fiber was revealed. High levels of energy, macronutrients, and sodium, and low levels of calcium and fiber; therefore, hospital menus may pose a risk of malnutrition. Hospital menus deviated from both national and international nutritional recommendations. These findings highlight the need for improved hospital planning and strategies to prevent hospital malnutrition.

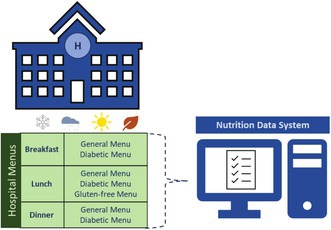

**Abbreviations**
ANOVA: Analysis of variance
EFSA: European Food Safety Authority
ESPEN: The European Society for Clinical Nutrition and Metabolism
FCS: Food Consumption Score
FDA: Food and Drug Administration
LIM: limited nutrient
MUFA: Monounsaturated fatty acids
NR: Nutrient rich
NRF: Nutrient Rich Food
SD: Standard deviation
SFA: Saturated fatty acids
TDG‐2022: Türkiye Dietary Guideline‐2022
USDA: Dietary Guidelines and US Department of Agriculture
WHO: World Health Organization

## 1 Introduction

One of the key elements that determines the health of a population is their nutrient patterns (Hu 2002). An adequate and balanced diet is considered one of the most critical factors in maintaining health (TDG 2022). Many scientific studies have shown how vital a proper and balanced diet, which can also be expressed as a healthy diet, is in preventing chronic diseases (Afshin et al. 2019). Malnutrition weakens the immune system and increases infection susceptibility (Tsuboyama‐Kasaoka and Purba 2014). A weakened immune system has many negative consequences, including slower wound healing, muscle loss, extended hospital stays, increased treatment costs, and higher mortality rates (Barker et al. 2011). It may develop negatively due to malnutrition in patients with acute or chronic diseases (Tappenden et al. 2013). Up to 40% of hospitalized patients are affected by disease‐related malnutrition (Tarantino et al. 2022). In addition, patients' nutrient status may be further compromised by food services that do not fully meet patients' needs (Allard et al. 2015, 2016; McCullough and Keller 2018; Rinninella et al. 2019).

Metabolic stress and increased activity of different metabolic pathways in pathological processes due to disease symptoms may also increase energy, macro‐ and micronutrient, and fiber requirements (Evans et al. 2008; Muscaritoli et al. 2010; Tappenden et al. 2013). Malnutrition due to illness may result in an increase in the rate of hospitalization or readmission due to prolonged treatment (Norman et al. 2008; Tappenden et al. 2013). Long‐term hospitalization is one of the most important causes of malnutrition. Hospital menus are unsuitable for patient needs, and patients' prejudice against these menus triggers malnutrition (Korfalı et al. 2009). In addition, it is essential for sustainability that the dishes on the menu are suitable for the season and that local products are preferred (Chatzipavlou et al. 2024). On the other hand, the inclusion of ultra‐processed foods in the hospital menu (about 25% of the total energy) shifts away from the sustainability concept (Detopoulou and Panoutsopoulos 2023). The concept of sustainable nutrition was first proposed by Gussow and Clancy, who argued that sustainability is vital for a healthy diet (Gussow and Clancy 1986). The Food and Agriculture Organization (FAO) developed a further definition for “sustainable diets” as “low environmental impact diets that contribute to food and nutrition security and healthy lives for present and future generations” (Iannetta et al. 2010). Later, the World Health Organization (WHO) and FAO published a guideline on the environmental sustainability of a healthy diet to support life and health (WHO 2019). Sustainable nutrition briefly refers to a diet with a low ecological impact that contributes to food security and health.

Hospital menus are the only food source for hospitalized patients. Patients unfamiliar with hospital menus may decrease their nutrient intake (Greig and Garcia 2016). According to statistical data from a study, approximately 58% of hospitalized patients did not consume all the food served to them (Kontogianni et al. 2020). In such cases, patients at risk of malnutrition have higher morbidity and mortality compared to those without this risk (Felder et al. 2015; Tangvik et al. 2014; Thibault et al. 2011). It is also inevitable that healthcare expenditures will increase (Khalatbari‐Soltani and Marques‐Vidal 2015). These reasons suggest that hospital menus should contain energy, macro‐ and micronutrients, and fiber at levels that will meet the nutrient needs of patients during their hospital stay (Trang et al. 2015). The European Society for Clinical Nutrition and Metabolism (ESPEN) advocates for a structured and patient‐oriented strategy in hospital menu planning, taking into account individual nutritional status, dietary limitations, and clinical requirements (Thibault et al. 2011). This guideline highlights that therapeutic diets—such as gluten‐free, low‐sodium, and diabetic diets—are frequently associated with insufficient energy intake. Accordingly, they recommend close monitoring of energy and nutrient distribution across different meals and seasons during the planning process. This perspective is essential for establishing hospital nutrition services that are personalized, nutritionally adequate, and sustainable.

Appropriate nutrient care in healthcare has been identified as a human right. WHO emphasizes that increased patient food and nutrient care investment can save lives (Wells et al. 2020). However, a previous study in England reported that most hospital menus did not meet patients' energy and protein needs (Pullen et al. 2018). Other studies in Spain and Greece reported that all hospital menus were inadequate in providing magnesium, vitamins E and D (Barcina‐Pérez et al. 2023; Detopoulou, Al‐Khelefawi, et al. 2022). On the other hand, it was stated that some menus provide minerals such as calcium, potassium, zinc, and copper, and also meet the energy and protein needs of patients (Barcina‐Pérez et al. 2023). Planning menus to provide adequate energy, macro‐ and micronutrients, and fiber will fulfill patients' needs for nutrients during their stay in the hospital and help prevent problems caused by malnutrition (Kirkland et al. 2013). Guidelines for hospital food service/menu planning have been developed in various countries (such as Sweden, Britain, Scotland, Canada, and the USA) (Kim et al. 2010; Pullen et al. 2018; Sorensen et al. 2021; The Scottish Government 2016; Wilandh et al. 2025), but there is no such guideline in Türkiye. However, although there are guidelines for the nutrient substance of hospital menus, investigations are needed to determine the extent to which hospital menus comply. It is unknown whether hospitals comply with these basic rules for healthy eating and whether the menus have a sufficient nutrient profile to meet the growing demand for nutrients acute patients need (Trang et al. 2015).

Türkiye Dietary Guideline‐2022 (TDG‐2022) provides national nutrition daily intake recommendations that support environmental sustainability while ensuring adequate and balanced nutrition for individuals (TDG 2022). The Food and Drug Administration (FDA) supports adopting sustainable practices in food production and promoting healthy eating habits (FDA 2013). The European Food Safety Authority (EFSA) informs policymakers with scientific data to create sustainable nutrition systems within the food safety framework (EFSA 2017). TDG‐2022 was compared with FDA and EFSA, and the recommendations for average daily energy, macro‐ and micronutrient levels are given in Table 1. According to the ESPEN guideline, energy requirements in hospital diets should be calculated as 30 kcal per kilogram of body weight. The recommended macronutrient distribution, expressed as a percentage of total energy intake, is 45%–50% for carbohydrates, 35%–40% for fat, and 20%–25% for protein (Thibault et al. 2011).

**TABLE 1:** Daily recommended average daily energy, macro‐ and micronutrient levels according to TDG‐2022, FDA ve EFSA.

	TDG‐2022	EFSA	FDA
Energy (kcal)	1599–1334 (M) 1276–1073 (F)	2696–3832 (M) 1815–2579 (F)	2000
Carbohydrate (g)	130	334–574.8 (M) 204.2–386.9 (F)	300
Fat (g)	60–89	74.9–106.4 (M) 50.4–71.6 (F)	65
Protein (g/d)	1.04 (g/kg/d)	67–114 (M) 59–102 (F)	56 (M) 46 (F)
Omega 3 (mg/d)	250	250	0.6–1.2
Saturated Fatty Acids	< 10% of daily energy	As low as possible	≤ 20 g/day (based on 2000 kcal)
Dietary fiber (g/d)	25	25	38 (M) 25 (F)
Vitamin A (μg)	750 (M) 650 (F)	570 (M) 490 (F)	900
Vitamin D (μg)	15	15	20
Vitamin E (mg)	13 (M) 11 (F)	13 (M) 11 (F)	15
Thiamin (mg)	0.4	0.6–1.0 (M) 0.5–0.7 (F)	1.2
Riboflavin (mg)	1.6	1.3	1.3
Niacin (mg)	6.6	12.3–17.5 (M) 8.3–11.8 (F)	16
Pyridoxine (mg)	1.7 (M) 1.6 (F)	1.5 (M) 1.3 (F)	1.7
Folate (μg)	330	250	400
Cobalamin (μg)	4	4	2.4
Vitamin C (mg)	110 (M) 95 (F)	90 (M) 80 (F)	90
Sodium (mg)	2000	2000	=2300
Potassium (mg)	3500	3500	4700
Calcium (mg)	950–1000	750	1300
Magnesium (mg)	350 (M) 300 (F)	350 (M) 300 (F)	420
Phosphorus (mg)	550	550	1250
Iron (mg)	11 (M) 16 (PREMF) 11 (POMF)	6 (M) 7 (PREMF) 6 (POMF)	18
Zinc (mg)	9.4–16.3 (M) 7.5–12.7 (F)	7.5–12.7 mg	11
	(depends on sex and phytate intake)	(depends on sex and phytate intake)	

Abbreviations: F, Female; M, Male; POMF, post‐menopause female; PREMF, pre‐menopause female.

In a study, three meal menu plans of five hospitals from different regions (Central Anatolia, Aegean, Mediterranean, Marmara, and Southeastern Anatolia) in Türkiye during one month (October 2022) were compared (Aytekin‐Sahin et al. 2023). This study stated that the nutrient profiles were not calculated based on a single portion as a limitation. No hospital menu standard or national accreditation is applied in hospitals in Türkiye (Oruçoğlu et al. 2024). In the study mentioned above (Aytekin‐Sahin et al. 2023), examining the nutrient profile of only one month's menu does not constitute a sufficient source of information about the nutrient profile of the menus of hospitals in Türkiye. According to the data of the General Directorate of Meteorology for Istanbul (2024) in 2023, the average winter temperature was recorded as 5.5°C, spring 12.8°C, summer 24.7°C, and autumn 17.4°C (MGM 2024). Since these temperature differences can affect the seasonal nutritional preferences and energy requirements of individuals, it is important to evaluate menus according to the seasons (Fujihira et al. 2023).

Menu planning is implemented within the hospital food services or the contracted food companies' criteria. It is essential to evaluate the nutrient profile of hospital diets to ensure optimum dietary intake (The Scottish Government 2016). Food and nutrient intake measurement is needed in this context, especially in hospitals. Additionally, a few studies have evaluated the nutrient profile of menus for patients in different countries (Aytekin‐Sahin et al. 2023; Thibault et al. 2011; Trang et al. 2015; Barcina‐Pérez et al. 2023; Detopoulou, Panoutsopoulos, et al. 2022). A retrospective study aimed to evaluate the nutritional profiles of various menus served in a hospital in Istanbul, Türkiye, in terms of energy, macro and micronutrients, and fiber, by making seasonal comparisons on a meal basis, and to examine the potential of these menus to pose a risk of malnutrition. The results of the nutrient profile were assessed according to FDA, EFSA, and TDG‐2022 requirements (EFSA 2017; FDA 2013; TDG 2022).

## 2 Methods

### 2.1 Study Design

**FIGURE 1:**
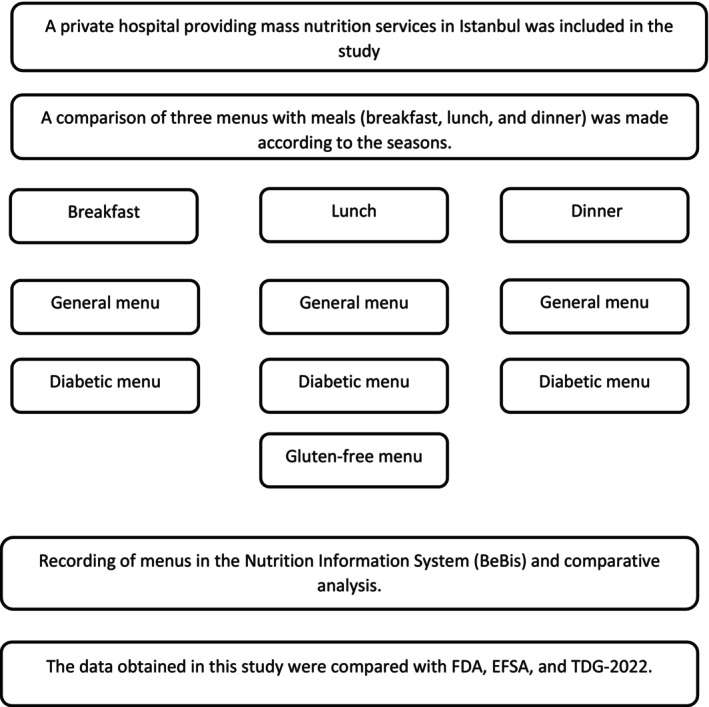
The flow diagram of the present study is given.

Data was obtained retrospectively from the hospital menu, which consists of three menus (general, diabetic, and gluten‐free menus) for a year (Istanbul, Türkiye). A comparison of three menus with meals (breakfast, lunch, and dinner) was made according to the seasons. Four months were chosen to represent each season (January, April, July, and October), and were included in this study to monitor seasonal changes in nutrient profile. Only the general and special menus were compared if only lunch meals were offered for the gluten‐free menu in the hospital. The flow diagram of the present study is given in Figure 1.

### 2.2 Dietary Data

All menus include three main meals: breakfast, lunch, and dinner. Examples of meal‐based general, diabetic, and gluten‐free menus according to seasons are given in the Supporting Information S1. The content of all menus is different from one another. Hospital food services offer four types of bread alternatives, including white, whole wheat, rye, and sourdough, at each meal. Individuals are allowed to choose up to two (100 g) according to their preferences.

The breakfast meal on the general menu includes cheese, olives, honey or jam, butter, chocolate or tahini molasses, and bread. It was observed that, unlike the general menu, diabetic products such as diabetic jam varieties and diabetic halva were included in the diabetic menu breakfast meals. The gluten‐free breakfast menu was similar to the general menu in terms of its contents, including cheese, olives, boiled eggs, and fresh vegetables. However, gluten‐free bread was not offered at the hospital.

The hospital's lunch and dinner consist of four table d'hôtes. The lunch and dinner meals in the general menu consist of: first bowl of soup, second bowl of red meat, chicken, legumes, third bowl of rice pilaf, bulgur pilaf, pasta, fourth bowl of fruit, salad, dessert, compote, ayran, yogurt, and tzatziki. The menu for diabetic patients consists of: first bowl of soup, second bowl of chicken and legumes, third bowl of bulgur pilaf, whole wheat pasta, vegetable dishes with olive oil, fourth bowl of fruit, salad, ayran, and tzatziki. The gluten‐free menu consists of: first bowl of soup, second bowl of meat and chicken, third bowl of salad, olive oil and potato rice and vegetable dishes, fourth bowl of compote, ayran, salad, and tzatziki. In the diabetic menu, unlike in other menus, it is not stated whether snacks will be provided or not.

### 2.3 Nutrient‐Rich Food 20.3 Index and Limited Nutrients Subscores

The Nutrient‐Rich Food 20.3 (NRF20.3) index was developed to identify healthy, affordable foods and food groups (Drewnowski 2009). In this case, foods recorded in the 2005 Dietary Guidelines and the US Department of Agriculture (USDA) were scored using the NRF index. The NRF index can be calculated per 100 kcal based on testing, validation, and other processes. In the present work, the nutrient value of hospital menus was determined using the NRF20.3 index score (Drewnowski 2009). The NR20 (nutrient‐rich food) was represented by protein, dietary fiber, monounsaturated fat (MUFA), linoleic acid, α‐linolenic acid, fish fatty acids, vitamin A, vitamin C, vitamin D, vitamin E, thiamin, riboflavin, pyridoxine, cobalamin, folate, calcium, iron, potassium, magnesium, and zinc.

The components of the LIM3 subscores are saturated fat, added sugar, and sodium. The NRF20.3 index score was found by the formula NR20 minus LIM3. Higher NRF20.3 index scores indicate higher nutrient density per 100 kcal; thus, menus with a high NRF20.3 index score have a healthier dietary pattern than those with a low NRF20.3 index score. Sweets, sugar, and beverages scored lowest on the NRF index, whereas fruit, vegetables, dry beans and legumes, eggs, meat, poultry, and fish scored highest (Drewnowski 2009).

### 2.4 Data Analysis

Food contents in the menus were calculated using the BeBIS Nutrition Data System for Research version 9.0 (Pacific Company, Istanbul, Türkiye). This scientific software program analyzes over 20,000 foods and more than 130 nutrients (BeBiS 2024). The content of the hospital menus was recorded as energy intake, macronutrients (carbohydrate, protein, and lipids), micronutrients (retinol, carotene, A, D, E, K, B1, B2, B3, B5, B6, B7, B9, B12, and C vitamins, Na, Ca, K, Cu, Fe, Mg, Zn, and P elements), dietary fiber, and the glycemic index. Additionally, data obtained in this study were compared to determine nutrient adequacy according to FDA, EFSA, and TDG‐2022 (EFSA 2017; FDA 2013; TDG 2022).

### 2.5 Statistical Analysis

Descriptive statistics were presented with mean ± standard deviation (SD). Two‐way ANOVA and one‐way ANOVA test analyses were applied to investigate whether there were differences in the menus' nutrient profiles. The Bonferroni test was used as a multiple comparison test. SPSS software (IBM Corp. Released 2021. IBM SPSS Statistics for Windows, Version 28.0. Armonk, NY: IBM Corp) was used for statistical analysis of the data. Results were evaluated in a 95% confidence interval and statistically at a *p* < 0.05 significance level.

## 3 Results

In this study, data obtained from the menus consisting of three daily meals (breakfast, lunch, and dinner) for one year, including general, gluten‐free, and diabetic menus of a hospital, were retrospectively analyzed. Determining bread consumption was outside the purpose of the study and could not be analyzed because it depended on the preferences of individuals.

Seasonal comparisons of the menus and associated meals are presented in Table 2. In this study, data obtained from one‐year, three‐day meal menus (including general, gluten‐free, and diabetic menus) in a hospital were retrospectively analyzed. Seasonal comparisons of menus and related meals are presented in Table 2. The analysis of energy distribution across different seasonal menus revealed notable variations between general and diabetic menus. In the winter season, breakfast contributed 12.30% and 14.60% of total daily energy, while lunch provided 39.74% and 41.71%, and dinner accounted for 47.56% and 43.69% in the general and diabetic menus, respectively. In spring, breakfast energy contributions increased to 23.65% for the general menu and 17.72% for the diabetic menu. Lunch and dinner energy shares in this season were 38.95% and 37.40% for the general menu, and 42.54% and 39.74% for the diabetic menu. During summer, breakfast comprised 21.71% of total energy in the general menu and dropped to 12.13% in the diabetic menu. Lunch provided 40.86% and 42.72%, while dinner offered 37.43% and 45.15%, respectively. In the autumn season, breakfast contributed 21.97% and 12.30% of daily energy in the general and diabetic menus, while lunch accounted for 39.66% and 39.74%, and dinner for 36.37% and 47.56%, respectively.

**TABLE 2:** Seasonal comparisons of menus and related meals.

	Winter	Spring	Summer	Autumn
Energy (kcal)	1621.64	1634.24	1663.07	1658.89
Carbohydrate (g)	161.56	158.67	161.45	169.13
Fat (g)	74.14	79.95	78.5	76.77
Protein (g/d)	72.78	67.25	73.18	68.27
Omega 3	1.97	2.88	2.08	2.02
Saturated Fatty Acids	32.06	33.45	32.62	32.84
Dietary fiber (g/d)	18.66	17.62	18.23	18.19
Vitamin A (μg)	1458.05	1655.26	1472.86	1541.14
Vitamin D (μg)	4.25	5.23	4.88	5.03
Vitamin E (mg)	8.96	10.74	10.83	10.73
Thiamin (mg)	0.79	0.91	0.84	0.84
Riboflavin (mg)	1.25	1.27	1.37	1.31
Niacin (mg)	22.05	20.62	20.77	19.17
Pyridoxine (mg)	1.46	1.41	1.49	1.41
Folate (μg)	271.01	284.77	290.36	286.05
Cobalamin (μg)	5.47	5.73	6.07	5.32
Vitamin C (mg)	106.03	127.85	115.81	108.05
Sodium (mg)	4333.28	4890.75	4350.4	4300.27
Potassium (mg)	2369.13	2469.3	2507.55	2283.52
Calcium (mg)	601.58	623.41	592.76	573.37
Magnesium (mg)	250.97	245.83	253.02	251.6
Phosphorus (mg)	1051.25	1010.22	1052.6	1039.43
Iron (mg)	10.12	10.14	10.84	10.67
Zinc (mg)	12.44	11.75	12.71	12.2

In this context, the average energy intake was approximately 1600 cal per season. According to TDG‐2022, the recommended daily energy intake varies by gender, ranging from 1334 to 1599 kcal for men and 1073 to 1276 kcal for women. According to EFSA recommendations, 2696–3832 cal are for men and 1815–2578 cal for women. The FDA recommends 2000 cal for both genders. The daily protein content of general menus was found to be between 67 and 73 g. According to TDG‐2022, daily protein intake is calculated as 1.04 g/kg/day per kg. EFSA recommends a slightly lower value of 0.83 g/kg/day, while the FDA recommends a daily intake of 0.8 g/kg/day, which equates to around 50 g for an average adult consuming a 2000‐cal diet. Additionally, NRF20.3 scores were found to be statistically insignificant at breakfast, lunch, and dinner in the seasons examined (*p* < 0.05) (Tables 3, 4, 5).

**TABLE 3:** Comparison of energy, nutrient intakes, and NRF 20.3 index scores across seasons in the breakfast meals on the general and diabetic menus.

Dietary intake	Winter		Spring		Summer		Autumn		*p*
	General	Diabetic	General	Diabetic	General	Diabetic	General	Diabetic	
Energy (kcal)	314.23 ± 34.6^bej^	200.00 ± 13.3^acg^	386.49 ± 82.3^bfk^	223.93 ± 40.3^adh^	361.05 ± 33.4^ef#^	176.85 ± 1.6^cdΔ^	364.52 ± 36.8^jk#^	176.85 ± 1.6^ghΔ^	< 0.001**
Carbohydrate (g)	24.39 ± 8.3	7.45 ± 1.52	35.18 ± 11.2	8.96 ± 3.0	25.55 ± 4.4	6.90 ± 1.0	25.75 ± 4.6	6.90 ± 1.0	0.350
Fat (g)	18.69 ± 2.4^bej^	14.03 ± 1.3^acg^	21.43 ± 7.0^bfk^	15.27 ± 2.92^adh^	22.08 ± 2.6 ^efl^	10.26 ± 0.2^cdΔ^	22.60 ± 2.4^jkl^	10.26 ± 0.2^ghΔ^	< 0.001**
Protein (g)	11.19 ± 2.3	10.65 ± 2.1	12.12 ± 3.8	11.88 ± 0.6	13.79 ± 1.6	13.25 ± 0.6	13.32 ± 1.2	13.25 ± 0.6	0.461
Fiber (g)	2.55 ± 0.5	1.08 ± 0.21	3.25 ± 1.4	1.56 ± 0.8	2.70 ± 0.6	1.93 ± 0.9	2.66 ± 0.5	1.93 ± 0.9	0.054
Cholesterol (mg)	130.57 ± 100.6^bej^	202.98 ± 66.7^acg^	135.08 ± 107.3^bfk^	231.14 ± 7.6^adh^	230.50 ± 4.8^efl^	218.3 ± 1.2^cdΔ^	230.29 ± 3.5^jkl^	218.3 ± 1.2^ghΔ^	< 0.001**
VITAMINS									
Vitamin A (μg)	441.11 ± 32.2	174.89 ± 28.0	562.98 ± 395.8	217.67 ± 59.8	496.73 ± 51.3	177.56 ± 65.5	491.23 ± 43.0	177.56 ± 65.5	0.275
Vitamin D (μg)	0.92 ± 0.5^bej^	1.16 ± 0.3^acg^	0.98 ± 0.6^bfk^	1.31 ± 0.0^adh^	1.41 ± 0.1^efl^	1.10 ± 0^cdΔ^	1.40 ± 0.1^jkl^	1.10 ± 0^ghΔ^	< 0.001**
Vitamin E (mg)	2.49 ± 0.9	1.12 ± 0.3	3.10 ± 1.7	1.54 ± 0.5	3.57 ± 0.74	1.53 ± 0.4	3.66 ± 0.6	1.53 ± 0.4	0.075
Thiamine (mg)	0.09 ± 0.0	0.08 ± 0.0	0.13 ± 0.8	0.10 ± 4.2	0.13 ± 0.1	0.10 ± 0	0.14 ± 0.1	0.10 ± 0.0	0.132
Riboflavin (mg)	0.28 ± 0.1	0.37 ± 0.1	0.32 ± 0.1	0.43 ± 0.0	0.41 ± 0.1	0.45 ± 0.1	0.41 ± 0.1	0.45 ± 0.1	0.808
Niacin (mg)	2.24 ± 0.7^bej^	1.82 ± 0.1^acg^	2.86 ± 0.9^bfk^	2.03 ± 0.3^adh^	2.21 ± 0.8^efl^	2.43 ± 0.4^cdΔ^	2.15 ± 0.6 ^jkl^	2.43 ± 0.4^ghΔ^	< 0.001**
Pyridoxine (mg)	0.17 ± 0.1	0.10 ± 0.0	0.24 ± 0.1	0.13 ± 0.0	0.25 ± 0.1	0.15 ± 0.1	0.26 ± 0.05	0.15 ± 0.1	0.663
Folate (μg)	42.99 ± 11.9^nrw^	46.65 ± 9.4^mot^	58.97 ± 24.1^nsy^	59.55 ± 19.0^mpu^	61.02 ± 15.1^rsz^	95.79 ± 19.7^opv^	62.30 ± 12.0^wyz^	95.77 ± 19.6^tuv^	0.001*
Cobalamin (μg)	0.82 ± 0.2	1.04 ± 0.2	0.91 ± 0.3	1.12 ± 0.0	1.00 ± 0.2	1.15 ± 0.1	0.96 ± 0.2	1.15 ± 0.1	0.904
Vitamin C (mg)	13.55 ± 7.6^bej^	0.80 ± 1.8^acg^	19.95 ± 12.7^bfk^	7.59 ± 10.6^adh^	15.80 ± 8.8^ef#^	12.62 ± 11.3^cdΔ^	14.94 ± 8.1^jk#^	12.62 ± 11.3^ghΔ^	< 0.001**
MINERALS									
Potassium (mg)	261.23 ± 119.9^nrw^	134.45 ± 51.4^mot^	388.26 ± 215.5^nsy^	231.22 ± 150.0^mpu^	326.89 ± 117.1^rsz^	311.00 ± 159.5^opΔ^	332.38 ± 105.0^wyz^	311.00 ± 159.5^tuΔ^	0.006*
Calcium (mg)	177.64 ± 32.8^bej^	161.13 ± 8.3^acg^	200.64 ± 23.4^bfk^	169.77 ± 8.7^adh^	173.02 ± 47.8^efl^	232.20 ± 9.3^cdi^	164.84 ± 47.1^jkl^	232.19 ± 9.3^ghi^	< 0.001**
Magnesium (mg)	31.13 ± 7.2	14.90 ± 1.9	38.68 ± 13.8	20.43 ± 7.1	35.48 ± 6.7	23.06 ± 7.4	35.00 ± 6.2	23.06 ± 7.4	0.603
Iron (mg)	1.30 ± 0.3^nrw^	1.03 ± 0.2^mot^	1.58 ± 0.5^nsy^	1.29 ± 0.2^mpu^	1.87 ± 0.3^rsz^	1.31 ± 0.2^opΔ^	1.87 ± 0.3^wyz^	1.31 ± 0.2^tuΔ^	0.035*
Zinc (mg)	1.53 ± 0.3	1.44 ± 0.2	1.77 ± 0.4	1.53 ± 0.0	1.66 ± 0.4	1.55 ± 0.1	1.61 ± 0.4	1.55 ± 0.1	0.737
Phosphorus (mg)	188.57 ± 45.4^bej^	203.77 ± 28.4^acg^	223.68 ± 62.4^bfk^	222.86 ± 15.3^adh^	220.46 ± 40.2^efl^	288.13 ± 14.5^cdΔ^	225.89 ± 48.2^jkl^	288.13 ± 14.5^ghΔ^	< 0.001**
LIPIDS									
Omega 3	0.39 ± 0.0	0.10 ± 0.0	0.47 ± 0.3	0.15 ± 0.1	0.40 ± 0.0	0.15 ± 0	0.40 ± 0.0	1.10 ± 0.0	0.651
PUFA (g)	2.31 ± 0.6^nrw^	1.04 ± 0.2^mot^	2.87 ± 2.2^nsy^	1.25 ± 0.3^mpu^	2.78 ± 0.6^rsz^	1.05 ± 0.1^opΔ^	3.07 ± 0.8^wyz^	1.05 ± 0.1^tuΔ^	0.001*
MUFA (g)	6.58 ± 1.3^bej^	4.31 ± 0.5^acg^	7.61 ± 3.1^bfk^	4.56 ± 0.8^adh^	7.30 ± 0.9^efl^	3.00 ± 0.0.0^cdΔ^	7.45 ± 0.8^jkl^	3.00 ± 0.0^ghΔ^	< 0.001**
LIM3									
Sucrose (g)	3.11 ± 4.2	0.01 ± 0.0	10.37 ± 6.4	0.12 ± 0.2	6.93 ± 5.7	0.05 ± 0.1	6.56 ± 5.0	0.05 ± 0.1	0.777
SFA (g)	8.73 ± 1.8^bej^	6.56 ± 1.1^acg^	9.74 ± 2.2^bfk^	7.08 ± 1.9^adh^	10.01 ± 1.6^efl^	4.05 ± 0.1^cdΔ^	10.04 ± 1.5^jk^	4.05 ± 0.1^ghΔ^	< 0.001**
Sodium (mg)	1469.54 ± 118.7^bej^	796.76 ± 20.8 ^acg^	1477.90 ± 249.5^bfk^	809.97 ± 21.8^adh^	1348.63 ± 204.7^efl^	834.76 ± 1.7^cdΔ^	1283.06 ± 229.9^jkl^	834.76 ± 1.7^ghΔ^	< 0.001**
NR20 Score	1851.14 ± 131.7	1023.71 ± 55.3	1952.53 ± 441.7	1094.98 ± 67.2	1803.89 ± 209.0	1122.46 ± 50.0	1737.18 ± 240.8	1122.58 ± 52.3	0.094
LIM3 Score	1481.40 ± 119.02^bej^	803.34 ± 20.1^acg^	1498.02 ± 249.0^bfk^	817.17 ± 22.8^adh^	1365.57 ± 208.2^efl^	838.87 ± 1.8^cdΔ^	1299.67 ± 236.5^jkl^	838.87 ± 1.8^ghΔ^	< 0.001**
NRF20.3 Score	369.74 ± 47.0	220.37 ± 37.9	454.51 ± 215.9	277.81 ± 62.1	438.31 ± 56.8	283.58 ± 48.2	437.51 ± 40.4	283.71 ± 50.5	0.896

*Note:* ** ***p*** **<** **0.001**; ^a^Winter–Spring diabetic, ^b^Winter–Spring general, ^c^Winter–Summer diabetic, ^d^Spring–Summer diabetic, ^e^Winter–Summer general, ^f^Spring–Summer general, ^g^Winter–Autumn diabetic, ^h^Spring–Autumn diabetic, ^i^Summer–autumn diabetic, ^j^Winter–Autumn general, ^k^Spring–Autumn general, ^l^Summer–Autumn general. * ***p* < 0.05**; ^m^Winter–Spring diabetic, ^n^Winter–Spring general, ^o^Winter–Summer diabetic, ^p^Spring–Summer diabetic, ^r^Winter–Summer general, ^s^Spring–Summer general, ^t^Winter–Autumn diabetic, ^u^Spring–Autumn diabetic, ^v^Summer–Autumn diabetic, ^w^Winter–Autumn general, ^y^Spring–Autumn general, ^z^Summer–Autumn general. ***p*** **>** **0.05**; ^Δ^Summer–Autumn diabetic, ^#^Summer–Autumn general.

Abbreviations: MUFA, Monounsaturated fatty acids; PUFA, Polyunsaturated fatty acids; SFA, Saturated fatty acids.

Breakfast meals included in the general and diabetic menus according to seasons are presented in Table 3. A significant difference was found between the seasons for energy, fat, cholesterol, vitamin D, niacin, vitamin C, calcium, phosphorus, MUFA, SFA, sodium nutrients, and LIM3 score in general and diabetic menus (*p* < 0.001). In general and diabetic menus, energy and MUFA amounts were higher in spring than in other seasons (*p* < 0.001). Fat (*p* < 0.001), SFA (*p* < 0.001), and PUFA (*p* = 0.001) amounts were higher in general menus in autumn and diabetic menus in spring compared to other seasons. Cholesterol and vitamin D levels were higher in general menus in summer and in diabetic menus in spring compared to other seasons (*p* < 0.001). It was observed that niacin, vitamin C, sodium nutrients, and LIM 3 values were higher in spring in general menus and in summer and autumn in diabetic menus compared to other seasons (*p* < 0.001). Potassium was higher in general menus in spring and in diabetic menus in summer and autumn compared to other seasons (*p* < 0.05). Calcium levels were determined to be higher in general menus in spring and diabetic menus in summer compared to other months (*p* < 0.001). While the amount of phosphorus was significantly higher in general and diabetic menus in autumn (*p* < 0.001), the amount of iron was significantly higher in both summer and autumn months (*p* < 0.05). Folate content was higher in general menus in autumn and diabetic menus in summer (*p* < 0.001).

Table 4 shows lunches of general, diabetic, and gluten‐free menus according to seasons. In diabetic lunch menus, a statistically significant difference was found in energy (*p* < 0.05) and carbohydrate (*p* < 0.001) values between spring and summer months. Energy and carbohydrate were found to be higher in summer months compared to other seasons. Fiber value was also found to be higher in winter and summer seasons (*p* < 0.05). This value was found to be higher in summer months compared to other seasons. Cholesterol content in lunches in gluten‐free menus was found to be statistically significant in winter–summer seasons (*p* < 0.05). Cholesterol levels in gluten‐free menus were found to be higher in winter months compared to other seasons. No significant difference was observed in general and diabetic meals (*p* > 0.05). Thiamine value in lunches in diabetic menus was found to be higher in summer seasons compared to other seasons (*p* < 0.05). Thiamine value in lunches of diabetic diet was also found to be significantly different between winter–spring and winter‐autumn (*p* < 0.05). It was observed that thiamine value was higher in summer months compared to other seasons. As a result of comparisons made in terms of vitamin C levels, a significant difference was found between general menus in spring and autumn seasons (*p* < 0.05). Diabetic menus showed a statistically significant difference between winter‐autumn and spring‐autumn seasons (*p* < 0.05). It was observed that vitamin C value was significantly higher in spring compared to other seasons in general and diabetic menus. In addition, lunch meals in diabetic menus showed statistically significant differences between winter and summer in terms of magnesium levels (*p* < 0.05). As a result of comparisons made in terms of magnesium levels in lunch diabetic menus, statistically significant differences were found between spring–summer, winter‐autumn (*p* < 0.05) and winter‐summer (*p* < 0.001) seasons. Iron mineral content of lunch meals in diabetic menus showed a statistically significant difference between winter‐autumn and spring–summer seasons (*p* < 0.05); also, a stronger significance was found between winter and summer seasons (*p* < 0.001). Magnesium and iron content of menus were higher in summer months compared to other seasons. Statistically significant differences were found between the LIM3 scores of general, diabetic, and gluten‐free menus examined in winter, spring, summer, and autumn seasons (*p* = 0.001). LIM3 was found to be higher in spring compared to other seasons.

**TABLE 4:** Comparison of energy, nutrient intakes, and NRF 20.3 index scores across seasons in the lunch meals on the general, diabetic, and gluten‐free menus.

Dietary intake	Winter			Spring			Summer			Autumn			*p*
	General	Diabetic	Gluten‐free	General	Diabetic	Gluten‐free	General	Diabetic	Gluten‐free	General	Diabetic	Gluten‐free	
Energy (kcal)	654.70 ± 140.2	571.20 ± 80.7^Δ^	573.08 ± 57.7	636.50 ± 132.7	537.63 ± 111.2^a^	560.05 ± 73.7	679.51 ± 119.6	622.88 ± 87.6^a^	545.60 ± 68.7	657.89 ± 134.3	571.34 ± 95.3^Δ^	553.41 ± 77.2	0.214
Carbohydrate (g)	68.40 ± 24.7	58.50 ± 21.3^Δ^	51.84 ± 13.8	60.59 ± 15.0	45.88 ± 19.7^i^	47.92 ± 16.3	68.80 ± 22.8	69.74 ± 20.2^i^	53.02 ± 17.8	67.18 ± 21.3	59.75 ± 21.5^Δ^	52.33 ± 16.1	0.215
Fat (g)	28.10 ± 8.2	23.90 ± 6.6	27.01 ± 4.9	30.68 ± 9.1	26.25 ± 7.0	28.43 ± 6.2	30.09 ± 9.0	23.98 ± 8.0	24.76 ± 7.3	29.67 ± 10.8	23.21 ± 6.2	25.57 ± 7.2	0.804
Protein (g)	30.40 ± 9.4	28.80 ± 7.8	28.94 ± 8.3	28.13 ± 8.3	27.87 ± 8.7	26.64 ± 7.5	31.75 ± 8.7	30.25 ± 6.4	26.10 ± 8.3	29.01 ± 8.8	29.23 ± 7.9	26.93 ± 8.1	0.712
Fiber (g)	8.30 ± 3.5	7.70 ± 2.3^bj#^	7.43 ± 2.7	7.42 ± 2.2	9.74 ± 3.1^#§▲^	7.61 ± 3.5	8.22 ± 3.3	11.5 ± 3.2^j§†^	7.09 ± 2.7	7.22 ± 2.8	9.80 ± 2.9^b▲†^	7.10 ± 2.7	0.001*
Cholesterol (mg)	123.70 ± 64.0	98.70 48.6	117.21 ± 57.8^c^	116.10 ± 75.5	90.02 ± 54.3	98.89 ± 54.2	97.42 ± 39.9	78.29 ± 35.4	77.60 ± 35.5^c^	101.71 ± 55.8	80.59 ± 43.2	81.91 ± 34.8	0.960
VITAMINS													
Vitamin A (μg)	509.00 ± 334.7	589.70 ± 393.8	520.16 ± 295.4	564.47 ± 417.1	595.90 ± 406.4	641.52 ± 478.7	483.20 ± 223.2	443.94 ± 255.1	570.23 ± 328.4	540.88 ± 449.1	466.14 ± 307.9	571.90 ± 322.0	0.764
Vitamin D (μg)	1.70 ± 2.2	1.20 ± 2.2	1.16 ± 1.5	2.20 ± 2.7	0.92 ± 1.2	1.26 ± 1.4	1.40 ± 2.3	1.28 ± 1.9	1.22 ± 1.7	2.18 ± 2.9	1.18 ± 1.5	1.43 ± 1.6	0.807
Vitamin E (mg)	2.80 ± 1.8	3.10 ± 2.2	2.55 ± 1.7	4.19 ± 3.1	2.96 ± 1.6	3.72 ± 2.4	3.94 ± 3.6	2.7 ± 1.5	3.51 ± 2.3	3.94 ± 2.9	2.80 ± 1.9	3.50 ± 2.2	0.417
Thiamine (mg)	0.40 ± 0.1	0.30 ± 0.1^dbj^	0.34 ± 0.1	0.38 ± 0.1	0.47 ± 0.2^d§▲^	0.37 ± 0.1	0.37 ± 0.1	0.55 ± 0.2^j§†^	0.34 ± 0.1	0.34 ± 0.1	0.47 ± 0.2^b▲†^	0.35 ± 0.1	0.001*
Riboflavin (mg)	0.40 ± 0.1	0.60 ± 0.2	0.49 ± 0.1	0.49 ± 0.2	0.54 ± 0.2	0.50 ± 0.24	0.48 ± 0.1	0.44 ± 0.1	0.45 ± 0.1	0.46 ± 0.2	0.46 ± 0.1	0.46 ± 0.1	0.128
Niacin (mg)	7.10 ± 2.8	7.70 ± 4.0	6.21 ± 1.8	6.55 ± 2.9	6.78 ± 2.8	7.07 ± 3.2	7.32 ± 3.7	7.91 ± 2.5	6.95 ± 4.2	6.65 ± 4.0	7.36 ± 3.3	6.91 ± 4.2	0.839
Pyridoxine (mg)	0.70 ± 0.2	0.70 ± 0.3	0.63 ± 0.1	0.60 ± 0.1	0.70 ± 0.2	0.65 ± 0.2	0.63 ± 0.2	0.64 ± 0.2	0.62 ± 0.2	0.59 ± 0.2	0.65 ± 0.2	0.63 ± 0.2	0.822
Folate (μg)	116.10 ± 47.4	132.50 ± 58.7	121.90 ± 43.3	127.35 ± 73.5	141.27 ± 73.3	139.54 ± 76.4	119.91 ± 62.8	117.66 ± 56.1	115.70 ± 49.6	102.63 ± 57.7	111.26 ± 52.1	117.49 ± 49.8	0.948
Cobalamin (μg)	2.50 ± 1.3	2.40 ± 1.1	2.64 ± 1.6	2.48 ± 1.2	2.33 ± 1.1	2.20 ± 1.1	2.70 ± 1.4	2.52 ± 1.2	2.55 ± 1.5	2.19 ± 1.2	2.22 ± 1.2	2.50 ± 1.4	0.896
Vitamin C (mg)	43.50 ± 25.2	71.30 ± 50.9^f#w^	62.08 ± 46.4	59.91 ± 34.7^e^	76.20 ± 45.2^g#§^	72.97 ± 41.1	46.10 ± 25.3	52.98 ± 38.2^w§†^	58.00 ± 35.2	36.89 ± 19.6^e^	43.09 ± 26.1^fg†^	58.21 ± 35.1	0.492
MINERALS													
Potassium (mg)	1110.50 ± 190.4	1079.30 ± 263.6	1195.96 ± 327.8	1092.48 ± 231.7	1219.42 ± 293.4	1209.46 ± 328.5	1141.11 ± 259.5	1105.75 ± 205.8	1137.49 ± 337.0	971.40 ± 262.4	1057.76 ± 230.0	1150.78 ± 318.0	0.375
Calcium (mg)	188.10 ± 83.8	258.10 ± 136.3^h#Δ^	219.70 ± 97.6	204.33 ± 96.7	249.36 ± 96.0^a#▲^	227.11 ± 100.5	200.25 ± 93.0	175.74 ± 73.4^ah†^	192.28 ± 92.0	215.50 ± 97.4	207.84 ± 89.2^Δ▲†^	193.99 ± 91.7	0.117
Magnesium (mg)	111.10 ± 26.2	106.90 ± 26.1^fj#^	103.53 ± 24.6	105.32 ± 26.1	117.45 ± 32.0^a#▲^	103.02 ± 31.9	113.38 ± 28.8	134.36 ± 22.4^aj†^	94.65 ± 17.8	107.36 ± 28.4	127.26 ± 31.2^f▲†^	96 ± 17.4	0.004*
Iron (mg)	4.80 ± 1.5	4.30 ± 1.2^fj#^	4.42 ± 1.3	4.55 ± 1.8	4.79 ± 1.5^a#▲^	4.29 ± 1.50	4.89 ± 1.9	5.33 ± 0.9^aj†^	4.01 ± 1.1	4.31 ± 1.5	5.03 ± 1.3^f▲†^	4.08 ± 1.1	0.056
Zinc (mg)	5.50 ± 2.1	6.20 ± 2.4	5.54 ± 2.6	5.18 ± 2.2	5.24 ± 1.4	4.84 ± 1.4	6.17 ± 2.3	6.68 ± 2.3	5.19 ± 2.3	5.27 ± 2.1	5.99 ± 2.2	5.33 ± 2.4	0.706
Phosphorus (mg)	425.80 ± 105.5	432.10 ± 96.4	402.68 ± 83.7	406.62 ± 116.7	453.58 ± 132.4	393.72 ± 104.2	434.72 ± 96.8	490.68 ± 104.3	366.48 ± 92.2	419.86 ± 102.4	475.23 ± 113.2	373.90 ± 87.0	0.207
LIPIDS													
Omega 3	0.90 ± 0.3	0.70 ± 0.4	0.60 ± 0.3	0.97 ± 0.4	0.65 ± 0.3	0.75 ± 0.4	0.88 ± 0.5	0.78 ± 0.4	0.71 ± 0.4	0.90 ± 0.4	0.81 ± 0.6	0.81 ± 0.5	0.634
PUFA (g)	4.30 ± 1.8	3.60 ± 1.7	3.49 ± 1.6	5.05 ± 2.3	3.49 ± 1.1	3.96 ± 1.4	5.20 ± 3.1	4.04 ± 1.5	3.92 ± 2.2	5.00 ± 2.7	3.77 ± 1.2	4.00 ± 2.2	0.933
MUFA (g)	10.20 ± 2.9	9.00 ± 2.4	10.34 ± 2.6	11.39 ± 3.3	10.5 ± 3.1	11.30 ± 2.8	10.85 ± 3.8	8.89 ± 3.3	9.55 ± 2.6	10.52 ± 3.9	9.46 ± 3.0	9.46 ± 3.0	0.770
LIM3													
Sucrose (g)	11.50 ± 15.5	12.60 ± 17.3	5.68 ± 11.5	7.46 ± 12.3	5.84 ± 10.2	6.39 ± 11.9	9.96 ± 15.6	12.68 ± 17.7	7.56 ± 13.6	12.97 ± 15.8	11.35 ± 18.2	11.82 ± 18.3	0.776
SFA (g)	11.80 ± 4.6	9.90 ± 2.9	11.51 ± 2.4	12.32 ± 4.5	10.65 ± 3.7	11.54 ± 3.4	11.61 ± 3.5	9.52 ± 3.2	10.59 ± 3.4	12.49 ± 5.8	9.37 ± 2.7	11.45 ± 3.5	0.938
Sodium (mg)	1507.40 ± 426.2	1294.50 ± 437.3	1446.82 ± 503.5	1676.87 ± 723.7	1598.88 ± 773.1	1720.51 ± 680.5	1558.31 ± 695.3	1384.37 ± 691.7	1468.50 ± 662.7	1593.64 ± 663.8	1614.12 ± 813.3	1483.09 ± 655.5	0.889
NR20 Score	2464.40 ± 487.3	2244.20 ± 540.9	2329.94 ± 559.1	2708.02 ± 824.5	2549.46 ± 842.2	2674.14 ± 725.4	2556.20 ± 803.5	2352.67 ± 779.0	2368.16 ± 721.5	2531.58 ± 759.0	2548.50 ± 898.9	2416.84 ± 660.6	0.093
LIM3 Score	1530.70 ± 424.5	1317.00 ± 432.2	1464.02 ± 503.2	1696.65 ± 723.0	1615.38 ± 773.7	1738.45 ± 671.0	1579.89 ± 692.9	1398.00 ± 685.9	1486.67 ± 660.9	1619.10 ± 676.1	1634.85 ± 818.1	1506.37 ± 656.3	0.001*
NRF20.3 Score	933.70 ± 143.4	927.20 ± 204.4	865.91 ± 152.2	1011.37 ± 196.9	934.07 ± 167.3	935.69 ± 225.3	976.31 ± 246.1	946.09 ± 202.7	881.49 ± 139.8	912.47 ± 201.3	913.64 ± 232.3	910.46 ± 245.7	0.783

*Note:* ** ***p*** **<** **0.001**; ^i^Spring–Summer diabetic, ^j^Winter–Summer diabetic. * ***p*** **< 0.05**; ^a^Spring‐Summer diabetic, ^b^Winter– Autumn diabetic, ^c^Winter–Summer Celiac, ^d^Winter–Spring diabetic, ^e^Spring–Autumn general, ^f^Winter–Autumn diabetic, ^g^Spring–Autumn diabetic, ^h^Winter–Summer diabetic. ***p* > 0.05**; ^Δ^Winter‐Autumn diabetic, ^#^Winter–Spring diabetic, ^§^Spring–Summer diabetic, ^▲^Spring–Autumn diabetic, ^†^Summer‐Autumn diabetic, ^w^Winter–Summer diabetic.

Abbreviations: MUFA, Monounsaturated fatty acids; PUFA, Polyunsaturated fatty acids; SFA, Saturated fatty acids.

The dinner meals of the general and diabetic‐specific menus by season are presented in Table 5. For the carbohydrate value in the dinner meal, a statistically significant difference was observed between seasons in the general and diabetic menus (*p* = 0.05). It was determined that the carbohydrate value in general and diabetic menus was higher in the autumn than in other seasons. For the magnesium value, a statistically significant difference was detected between seasons in the general and diabetic menus (*p* = 0.05). The magnesium level was higher in the general menu during the spring and the diabetic menu during the autumn compared to other seasons. At the dinner meals, a significant difference for phosphorus was observed in general and diabetic menus between seasons (*p* = 0.05). The phosphorus value in the general menu was higher in the winter season, while the diabetic menu was higher in the autumn season than in other seasons. No significant difference was found between the summer and autumn seasons for carbohydrate, magnesium, and phosphorus values at the dinner meals (*p* > 0.05). Furthermore, the NR20, LIM3, and NRF20.3 scores did not show any statistically significant variation (*p* > 0.05).

**TABLE 5:** Comparison of energy, nutrient intakes, and NRF 20.3 index scores across seasons in the dinner meals on the general and diabetic menus.

Dietary intake	Winter		Spring		Summer		Autumn		*p*
	General	Diabetic	General	Diabetic	General	Diabetic	General	Diabetic	
Energy (kcal)	652.65 ± 183.0	598.29 ± 126.3	611.23 ± 192.3	502.25 ± 140.5	622.5 ± 145.7	658.33 ± 184.3	636.48 ± 192.0	685.5 ± 218.0	0.117
Carbohydrate (g)	68.76 ± 29.2^acg^	59.73 ± 30.4^bei^	62.9 ± 27.1^adh^	44.09 ± 18.7^bfj^	67.09 ± 26.9^cdΔ^	79.31 ± 37.1^efk^	76.18 ± 39.3^ghΔ^	82.55 ± 42.8^ijk^	0.046*
Fat (g)	27.32 ± 10.0	26.45 ± 6.1	27.83 ± 11.4	23.92 ± 9.2	26.31 ± 5.6	24.11 ± 6.1	24.48 ± 6.9	24.82 ± 5.5	0.225
Protein (g)	31.13 ± 11.8	28.66 ± 8.8	27 ± 8.5	26.21 ± 8.2	27.62 ± 9.6	29.04 ± 10.8	25.93 ± 9.3	30.78 ± 9.9	0.351
Fiber (g)	7.80 ± 2.5	8.60 ± 2.7	6.94 ± 2.6	7.88 ± 2.1	7.29 ± 2.6	12.52 ± 6.9	8.29 ± 3.4	13.40 ± 7.5	0.240
Cholesterol (mg)	96.45 ± 45.7	90.91 ± 51.8	85.08 ± 32.1	73.73 ± 29.0	78.89 ± 34.1	67.50 ± 38.0	85.20 ± 53.6	74.15 ± 40.6	0.659
VITAMINS									
Vitamin A (μg)	507.91 ± 329.8	662.93 ± 373.9	527.8 ± 393.4	602.90 ± 378.8	492.91 ± 277.4	484.33 ± 271.3	509.02 ± 336.9	532.11 ± 345.9	0.267
Vitamin D (μg)	1.57 ± 2.2	0.79 ± 1.6	2.03 ± 2.8	1.47 ± 2.2	2.06 ± 2.0	1.60 ± 1.9	1.43 ± 2.2	1.10 ± 1.7	0.608
Vitamin E (mg)	3.66 ± 2.5	3.13 ± 1.5	3.44 ± 2.3	3.27 ± 2.1	3.31 ± 2.3	3.10 ± 2.1	3.11 ± 2.0	2.67 ± 1.4	0.964
Thiamine (mg)	0.34 ± 0.1	0.35 ± 0.2	0.39 ± 0.4	0.37 ± 0.1	0.33 ± 0.1	0.64 ± 0.5	0.35 ± 0.1	0.69 ± 0.5	0.090
Riboflavin (mg)	0.51 ± 0.2	0.49 ± 0.2	0.45 ± 0.2	0.47 ± 0.2	0.46 ± 0.1	0.48 ± 0.2	0.42 ± 0.2	0.51 ± 0.2	0.326
Niacin (mg)	12.65 ± 5.6	12.81 ± 4.3	11.20 ± 3.9	11.32 ± 3.5	11.22 ± 4.5	12.57 ± 4.8	10.36 ± 4.0	12.78 ± 4.1	0.577
Pyridoxine (mg)	0.61 ± 0.1	0.72 ± 0.2	0.56 ± 0.1	0.64 ± 0.1	0.59 ± 0.2	0.69 ± 0.2	0.55 ± 0.1	0.68 ± 0.1	0.851
Folate (μg)	111.89 ± 39.9	140.36 ± 68.8	98.44 ± 30.8	117.49 ± 57.2	109.41 ± 50.8	124.86 ± 64.4	121.10 ± 66.1	137.55 ± 50.1	0.926
Cobalamin (μg)	2.13 ± 1.5	2.45 ± 1.6	2.33 ± 1.3	2.17 ± 1.3	2.34 ± 1.4	2.2 ± 1.4	2.16 ± 1.2	2.34 ± 1.1	0.548
Vitamin C (mg)	48.94 ± 34.1	63.46 ± 34.5	47.98 ± 32.8	73.05 ± 37.0	53.9 ± 42.0	54.24 ± 39.0	56.21 ± 34.8	54.80 ± 34.2	0.091
MINERALS									
Potassium (mg)	997.36 ± 211.4	1133.29 ± 265.6	988.55 ± 349.3	1067.00 ± 212.0	1039.54 ± 231.2	1186.7 ± 283.3	979.73 ± 261.8	1222.48 ± 273.1	0.618
Calcium (mg)	235.87 ± 105.8	227.28 ± 119.4	218.43 ± 118.8	222.55 ± 94.4	219.48 ± 92.5	226.76 ± 95.0	193.03 ± 97.2	238.12 ± 113.6	0.480
Magnesium (mg)	108.77 ± 25.5^acg^	110.99 ± 30.5^bei^	310.82 ± 26.5^adh^	98.89 ± 24.0^bfj^	104.15 ± 23.6^cdΔ^	160.24 ± 61.5^efk^	109.24 ± 35.1^ghΔ^	168.5 ± 72.8^ijk^	0.004*
Iron (mg)	4.04 ± 1.3	4.38 ± 1.4	3.99 ± 1.3	3.97 ± 1.0	4.07 ± 1.3	5.98 ± 2.1	4.47 ± 1.6	6.46 ± 2.5	0.054
Zinc (mg)	5.43 ± 3.4	5.42 ± 2.1	4.8 ± 1.8	4.84 ± 1.9	4.87 ± 2.2	6.2 ± 2.5	5.31 ± 2.5	6.96 ± 3.1	0.395
Phosphorus (mg)	436.82 ± 106.2^acg^	418.41 ± 115.4^bei^	379.92 ± 95.4^adh^	393.02 ± 110.2^bfj^	397.40 ± 107.2^cdΔ^	537.02 ± 216.2^efk^	393.67 ± 121.5^ghΔ^	580.21 ± 241.0^ijk^	0.027*
LIPIDS									
Omega 3	0.70 ± 0.21	0.75 ± 0.2	1.42 ± 4.0	0.64 ± 0.3	0.78 ± 0.2	0.76 ± 0.2	0.70 ± 0.2	0.77 ± 0.2	0.139
PUFA (g)	4.21 ± 1.6	3.81 ± 1.3	5 ± 4.9	3.58 ± 1.8	4.08 ± 1.4	4.02 ± 1.2	3.78 ± 1.5	3.99 ± 0.8	0.129
MUFA (g)	9.95 ± 4.0	10.37 ± 3.7	9.64 ± 3.7	9.61 ± 4.2	9.89 ± 2.5	8.74 ± 2.7	8.95 ± 2.7	8.88 ± 2.4	0.174
LIM3									
Sucrose (g)	8.77 ± 15.3	9.29 ± 14.4	5.24 ± 6.9	3.89 ± 4.5	4.61 ± 6.4	9.09 ± 15.9	13.25 ± 17.9	12.55 ± 18.8	0.646
SFA (g)	11.50 ± 5.3	10.86 ± 3.0	11.38 ± 3.8	9.39 ± 3.8	10.99 ± 2.9	9.98 ± 3.1	10.3 ± 3.1	10.27 ± 2.6	0.283
Sodium (mg)	1356.32 ± 415.1	1396.96 ± 425.0	1735.97 ± 1759.0	1620.98 ± 717.84	1443.45 ± 392.4	1422.15 ± 418.1	1423.56 ± 721.0	1537.22 ± 808.6	0.899
NR20 Score	1660.32 ± 438.7	1700.84 ± 420.8	2207.11 ± 1813.5	2088.78 ± 830.6	1742.78 ± 394.1	1724.93 ± 418.0	1730.83 ± 717.1	1843.77 ± 803.4	0.943
LIM3 Score	1376.60 ± 416.4	1417.12 ± 426.8	1752.6 ± 1760.7	1634.27 ± 721.8	1459.06 ± 393.2	1441.22 ± 413.7	1447.12 ± 722.8	1560.05 ± 816.1	0.913
NRF20.3 Score	865.86 ± 190.3	951.27 ± 190.6	1124.84 ± 1586.1	871.40 ± 193.8	894.74 ± 192.1	967.30 ± 208.7	836.32 ± 202.8	995.54 ± 202.7	0.386

*Note:* * ***p* < 0.05**. ^a^Winter‐Spring general, ^b^Winter‐Spring diabetic, ^c^Winter–Summer general, ^d^Spring‐Summer general, ^e^Winter–Summer diabetic, ^f^Spring‐Summer diabetic, ^g^Winter–Autumn general, ^h^Spring–Autumn general, ^i^Winter–Autumn diabetic, ^j^Spring–Autumn diabetic, ^k^Summer‐Autumn diabetic. ***p* > 0.05**; ^Δ^Summer‐Autumn general.

Abbreviations: MUFA, Monounsaturated fatty acids; PUFA, Polyunsaturated fatty acids; SFA, Saturated fatty acids.

## 4 Discussion

In a retrospective study, three main meals from three different menus served throughout the year in a hospital were evaluated on standard portions. Within the scope of the study, nutritional profile analyses of each meal were carried out, representing four seasons, and seasonal differences were examined.

Hospital menus in Turkey do not have standardized energy and nutrient content (Oruçoğlu et al. 2024). Hospitalized patients and the staff consume at least one meal daily. The nutrient profile of menus plays a crucial role in supporting healthy eating (Aytekin‐Sahin et al. 2023; Trang et al. 2015). A study comparing five different hospital menus from four different geographic regions of Türkiye observed that although all menus contained higher energy, protein, fat, saturated fat, and sodium than the TDG‐2022 recommendations, their fiber and carbohydrate content was lower (Aytekin‐Sahin et al. 2023). When examining the menus according to the seasons, it was observed that, particularly in terms of the dairy group, vegetables, and fruit portions, the recommendations were consistently not met (Oruçoğlu et al. 2024; Wells et al. 2020). The study's overall menus have an energy content ranging from 1621 to 1663 kcal. According to TDG‐2022 (TDG 2022), daily energy intake was found to be high for both genders, while according to EFSA (EFSA 2017), it was low. In this study, the seasonal analysis of hospital menus revealed that the energy content was at its lowest in winter, the coldest season, and reached its peak during the summer months. A study conducted in five different hospitals reported that the average energy content (2171–2601 kcal) of one month's menus served in October exceeded the recommendations outlined in TDG‐2022 (Aytekin‐Sahin et al. 2023). In contrast, the current study found a seasonal average energy intake of approximately 1600 kcal, which corresponds to the lower limit of the TDG‐2022 guidelines. This finding suggests that the menus evaluated in our study were appropriately formulated, especially considering the low levels of physical activity often observed in hospitalized individuals. The average energy content of the general menus of three different hospitals in Canada was similar to our study (Trang et al. 2015). In another study, while the 14‐day basal, diabetic, and soft diets in the winter hospital menu adequately fulfilled the energy and protein requirements of patients on basal and soft diets, the energy intake of the diabetic diet was found to be inadequate (Barcina‐Pérez et al. 2023).

The daily protein content of general menus was found to be between 67 and 73 g. Daily protein intake for female patients was determined to be sufficient according to TDG‐2022, EFSA, and FDA reference values; however, insufficiency was observed for male patients, which may pose a potential risk of malnutrition in male patients. According to some studies, a protein‐rich breakfast may enhance satiety, potentially resulting in reduced protein intake during subsequent meals (Paddon‐Jones and Leidy 2014; Westerterp‐Plantenga et al. 2012); however, other evidence supports that consuming more protein in the morning contributes to higher overall daily protein intake (Verreijen et al. 2021). Ensuring 20 g of protein at each primary meal, breakfast included, may serve as a feasible method to minimize the risk of inadequate protein consumption (Koopmans et al. 2025). When calories and protein are high, portion sizes tend to increase, which can lead to plate waste. In studies conducted, a reduction in portion sizes has been associated with decreased plate waste (Freedman and Brochado 2010; Williams and Walton 2011). The wastage of hospital meals diminishes patients' daily intake of energy and protein, resulting in insufficient fulfillment of protein requirements, particularly among male patients (Barton et al. 2000). In another study, full‐sized and reduced portion‐sized main dishes were served; the food waste and energy intake and intakes of total fat, saturated fat, cholesterol, sodium, fiber, calcium, potassium, and iron were significantly lower compared to the full‐sized main dishes (Berkowitz et al. 2016). Food waste reduction is essential for sustainable food service systems in hospitals. According to TDG‐2022, the menus provided in this study were found to have a high energy content. Reducing portion sizes in menus will help prevent individuals from consuming excessive calories and reduce plate waste (Greig and Garcia 2016).

In a study comparing changes in the general menu over time, statistically significant differences were found in terms of NRF20.3 values (Günalan et al. 2024). In a study, the average Food Consumption Score (FCS) values for 14‐day hospital menus were reported as 23.1, 45.7, and 53.5 for breakfast, lunch, and dinner, respectively (Detopoulou, Panoutsopoulos, et al. 2022). In contrast, the NRF20.3 scores in our study, when calculated from annual averages, were 425.01 for breakfast, 958.46 for lunch, and 930.44 for dinner. Although these scores originate from different periods and are therefore not directly comparable, similar meal‐based ranking patterns (especially increasing lunch scores) suggest menu consistency in terms of variety and nutritional quality. However, no statistically significant difference was observed between the NRF20.3 scores. Furthermore, since FCS captures short‐term dietary assessment, NRF20.3 is based on annual average data; this temporal inconsistency should be taken into account when interpreting the results.

When the menus are examined seasonally, the proportion of energy obtained from SFA is approximately 18%. Cholesterol and SFA intake levels are relatively high according to TDG‐2022 and EFSA recommendations. It is recommended to limit SFA in the daily diet to less than 10% of total calorie intake to reduce cardiovascular diseases (Harcombe 2019). Replacing SFA‐containing foods in the diet with proteins, monounsaturated and polyunsaturated fats, and whole grain products may reduce the risk of cardiovascular disease (Briggs et al. 2017). The study recommendations are to reduce the amounts of cholesterol and SFA in the menus since hospitalized patients also consume the menus examined. Since hospitalized patients also consume the menus examined in the study, it is recommended to reduce sodium, cholesterol, and SFA. When the menus are examined seasonally, it is seen that more weight is given to the meat group, such as red meat and chicken; less weight is given to the dairy group, such as ayran and tzatziki; and less weight is given to the vegetable and fruit group. In addition, it was observed that the amount of fiber consumed through daily nutrition in the menus was low according to the TDG‐2022 and EFSA recommendations.

The intensive use of red meat in menus increases the carbon and water footprint (Aytekin‐Sahin et al. 2023; Jarmul et al. 2020). According to the Mediterranean diet and the hospital meal menus planned according to TDG‐2022, they have lower saturated fat and cholesterol content but contain higher dietary fiber (Oruçoğlu et al. 2024). Planning hospital menus according to the Mediterranean diet model and the TDG‐2022 recommendations is an essential strategy for preventing cardiovascular diseases. This approach aims to reduce the amount of cholesterol and SFA in the menus while increasing the fiber content. Studies show that reducing carbon and water footprints makes sustainable menus, such as the Mediterranean diet, more environmentally advantageous than traditional menus (Aytekin‐Sahin et al. 2023; Blas et al. 2019; Saleki et al. 2023).

High sodium intake and low potassium intake can lead to non‐communicable diseases such as hypertension, cardiovascular diseases, and stroke. High‐potassium diets prevent these diseases and maintain health (Farapti et al. 2022). One study shows that reducing dietary sodium intake in middle‐aged and older adults significantly lowered blood pressure, independent of antihypertensive medication use (Gupta et al. 2023). In another study, while moderate sodium intake was not associated with reducing cardiovascular disease risk, it supports the importance of increasing potassium and magnesium intake. Promoting foods rich in potassium and magnesium, such as vegetables, fruits, and dairy products, may reduce cardiovascular disease risk (Pickering et al. 2021). The study observed high sodium and phosphorus intake, while potassium, calcium, and magnesium levels were found to be low. This may be due to the use of ultra‐processed foods such as triangle cheese, tomato sauce dishes, and ready‐made soups in the menus offered (Detopoulou and Panoutsopoulos 2023). The menu provided may cause an increase in blood pressure in individuals consuming it. Hypertension can also lead to non‐communicable diseases such as cardiovascular diseases and stroke (Gupta et al. 2023). It is important to include food groups rich in calcium, potassium, and magnesium in hospital menu planning to prevent hypertension and cardiovascular diseases. The increased inclusion of dairy products, vegetables, and fruits in menus and larger portions could be significant in preventing various diseases.

In addition, a direct relationship could not be established between seasonal vitamin and mineral needs and menu contents in this study. For example, no special planning was found in the menus to meet the increasing vitamin D content due to less exposure to the sun in the winter months. Although vitamin and mineral contents were compared statistically between seasons, there is no data that would allow these contents to be compatible with characteristic needs. In the present study, only menus served in the hospital kitchen were evaluated, and individual consumption systems were not reached. There is no data on patient distribution, such as how many patients consumed the menus and for how long, the number of hospitalized patients, and the average hospitalization locations. Therefore, the consumption amounts per person of the foods in the menu could not be evaluated. The connections made regarding the impact of environmental relationships are also limited in potential content. On the other hand, this study cannot reflect the actual consumption levels and individual food supply rates; in addition to the above reasons, only the structural and content recording values of the menu are limited.

### 4.1 Study Limitations

The limitation of this study is that the menus were examined for a single hospital for 1 year. More hospital menus could have been compared. Also, the menus could have been reviewed at regular intervals instead of a specific period of 1 year.

## 5 Conclusions

As a result, it was observed that the nutritional profile of the hospital menus examined in the study did not resemble the recommendations of either national or international authorities, and there were imbalances in terms of nutritional intake. High levels of energy, carbohydrates, protein, fat, saturated fat, and sodium, and low levels of calcium and fiber in the menus in all seasons may increase the risk of nutrient deficiency for both genders. However, when the amount of protein in the menus is considered, it is seen that hospital menus pose a risk of malnutrition for men while being sufficient for women. In addition, the menus are incompatible with sustainable nutrition principles that can be improved in terms of individual health and environmental sustainability. Although a standardized planning approach is not widely applied in hospital menus in Turkey, the findings of this study show that the menus examined are unlikely to pose a risk of malnutrition.

## Author Contributions

**Beyza Mendes:** conceptualization (equal), data curation (equal), formal analysis (equal), investigation (equal), methodology (equal), visualization (equal), writing – original draft (equal), writing – review and editing (equal). **Ayse Gunes Bayir:** conceptualization (equal), formal analysis (equal), methodology (equal), supervision (equal), visualization (equal), writing – review and editing (equal). **Ayse Semra Aksoy:** data curation (equal), formal analysis (equal), investigation (equal), validation (equal), visualization (equal), writing – review and editing (equal). **Ozlem Toluk:** data curation (equal), formal analysis (equal), methodology (equal), writing – review and editing (equal).

## Conflicts of Interest

The authors declare no conflicts of interest.

## Supporting information


**Supporting Information S1** Examples of meal‐based general, diabetic, and gluten‐free menus according to seasons.

## Data Availability

The datasets generated and/or analyzed during the current study are available from the corresponding author upon reasonable request.
