# Supplementary material for: A Retrospective Study: Do Hospital Menus Carry a Risk of Malnutrition?
Source: Food Sci Nutr. 2025 Jul 29;13(8):e70669. doi: 10.1002/fsn3.70669 (PMC12305671; doi:10.1002/fsn3.70669)
Supplement: Supplementary file 1 — Supporting Information S1 Examples of meal‐based general, diabetic, and gluten‐free menus according to seasons. [file FSN3-13-e70669-s001.docx]

| **Meals** | **Menus** | **Winter** | **Spring** | **Summer** | **Autumn** |
| --- | --- | --- | --- | --- | --- |
| **Breakfast** | **General** | Tea  Soup  Black olives  White cheese  Boiled egg | Tea  Soup  Black olives  White cheese  Tomatoes  Cucumbers  Boiled egg  Pumpkin tart | Tea  Soup  Green olives  Cream cheese  Boiled egg  Tomatoes  Cucumbers | Tea  Soup  Black olives  White cheese  Jam  Boiled egg |
|  | **Diabetic** | Tea  Black olives  Boiled egg  Ezine cheese  Iceberg lettuce  Parsley | Tea  Black olives  Ezine cheese  Boiled egg  Tomatoes  Cucumbers | Tea  Black olives  Boiled egg  White cheese  Tomatoes-cucumbers | Tea  Black olives  Boiled egg  White cheese  Tomatoe-cucumbers |
|  | **Gluten-free** | n.d. | n.d. | n.d. | n.d. |
| **Lunch** | **General** | Lentil soup  Boiled beef  Vermicelli rice pilaf  Winter salad | Yogurt soup  Chickpea stew  Chicken rice pilaf  Ayran | Chicken broth soup  Chickpeas with meat  Vegetable rice pilaf  Hayrabolu dessert | Tomato soup  Chicken schnitzel  Pasta with tomato sauce  Ayran |
|  | **Diabetic** | Broccoli soup  Meatball stew  Spinach with olive oil  Yogurt | Broccoli soup  Meatball stew  Spinach with olive oil  Yogurt | Chicken broth soup  Chickpeas with meat  Whole wheat spaghetti  Ayran | Tomato soup  Grilled chicken  Whole wheat pasta  Seasonal salad |
|  | **Gluten-free** | Lentil soup  Boiled beef  Plain rice  Ayran | Yogurt soup  Chickpea stew  Chicken  Ayran | Chicken broth soup  Chickpeas with meat  Vegetable rice pilaf  Ayran | Tomato soup  Chicken schnitzel  Olive oil braised mixed vegetables  Ayran |
| **Dinner** | **General** | Ezogelin soup  Sour meatballs  Leek pastry  Spiced sherbet | Ezogelin soup  Hungarian goulash  Green beans in olive oil  Shekerpare (sugar cakes) | Soup  Meatballs with cheese  Pasta / boiled  Mixed salad | Red lentil soup  Eggplant moussaka  Pasta/boiled  Trileche(milk cake) |
|  | **Diabetic** | Tomato soup  Vegetable bouquet  Walnut vermicelli  Cacık | Ezogelin soup  Hungarian goulash  Green beans in olive oil  Fresh fruit | Soup  Meatballs with cheese  Bulgur pilaf  Mixed salad | Red lentil soup  Eggplant moussaka  Bulgur pilaf  Full-fat yogurt |
|  | **Gluten-free** | n.d. | n.d. | n.d. | n.d. |
